# Cervical thymic cysts in children: A case report

**DOI:** 10.1016/j.ijscr.2024.110483

**Published:** 2024-10-28

**Authors:** Nadia Romdhane, Dorra Chiboub, Selima Jouini, Ons Kharrat, Raja Jouini, Chiraz Mbarek

**Affiliations:** a ENT Department, Habib Thameur Hospital, Tunis, Tunisia; b Anatomopatholgy Department, Habib Thameur Hospital, Tunis, Tunisia

**Keywords:** Thymic, Cyst, Children, Cervical, Mass

## Abstract

**Introduction and importance:**

Cervical thymic cysts are rare benign lesions that rarely considered in the differential diagnosis of neck cysts in children. The correct diagnosis is often made after surgical excision and though determination of the specific histopathological findings of the thymic cyst.

**Case presentation:**

We report an observation of a cervical thymic cyst erroneously diagnosed preoperatively respectively as a cystic lymphangioma.

**Clinical discussion:**

The diagnosis is generally rectified after surgery by identifying the specific histopathological findings of the thymic cyst. Treatment is surgical.

**Conclusion:**

This diagnosis should be considered in front of any indolent lateral cervical mass.

## Highlights

• Cervical thymic cysts are rare benign lesions.
• Diagnosis is often post-operative.
• Treatment is surgical.
• Definitive diagnosis is histopathological.

## 1 Introduction

Cervical thymic cyst is a rare malformation of embryological origin [1] mostly. The cyst may appear at any level of the normal descent of the thymus from the angle of the mandible to the superior mediastinum [2]. It usually presents as a lateral painless mass and is often confused with the branchial cleft cyst, the cystic lymphangioma, or the thyroglossal duct cyst [3]. Treatment is surgical excision in all cases and the correct diagnosis is often made postoperatively [4]. In this paper, we report an observation of a cervical thymic cyst in 7-year-old boy, erroneously diagnosed preoperatively as a cystic lymphangioma. The work has been reported in line with the SCARE criteria [5].

## 2 Observation

We report the case of a 7-year-old boy, with no medical history, who presented with a left laterocervical swelling that evolved for one year, with no episodes of surinfection.

**Fig. 1:**
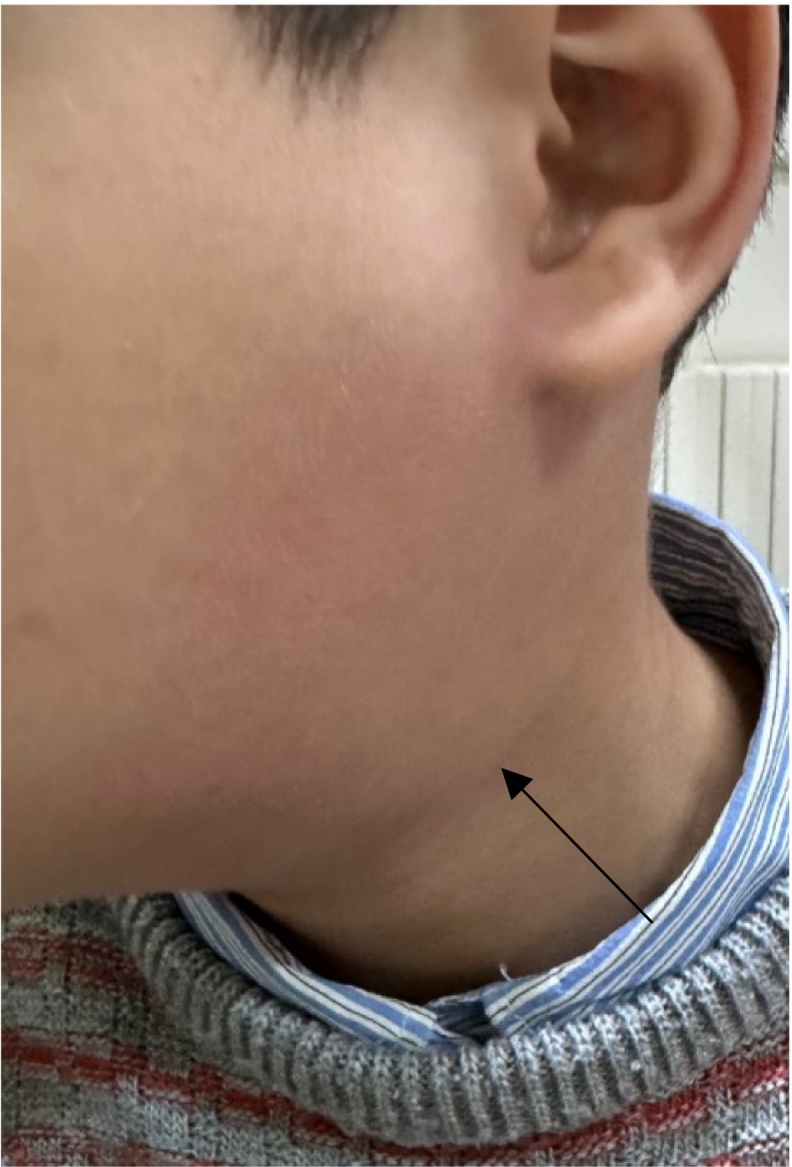
Left subdigastric swelling.

Physical examination found a 3 cm left soft subdigastric swelling (Fig. 1), associated with bilateral centimetric lymph nodes. Cervical ultrasound revealed an oval partitioned cystic mass, of the left carotid space, measuring 47 ∗ 30 mm, with a retrolaryngeal extension and intimate contact with the left common carotid artery and its internal and external branches. It evoked a cystic lymphangioma, a third branchial cleft cyst or a laryngocele.

**Fig. 2:**
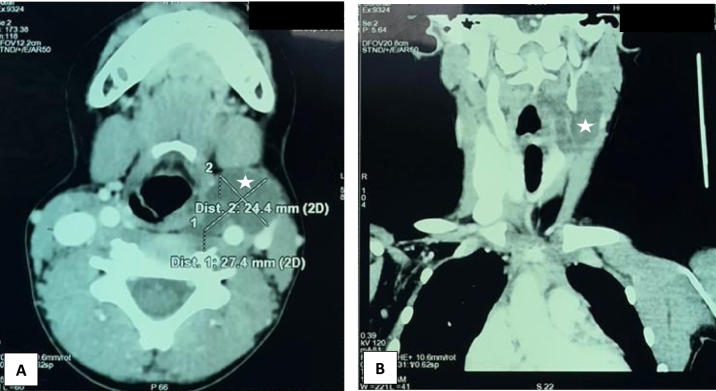
Cervical CT scan on axial (A) and coronal (B) images: Left latero-cervical multiloculated cystic mass (white star), measuring 27 ∗ 24 mm, with a mass effect on the oropharynx.

Cervical CT (Fig. 2) confirmed the presence of a multiloculated paraliquid left mass, measuring 50 ∗ 27 ∗ 24 mm. An extension to the retropharyngeal space, responsible for a mass effect on the oropharynx, with suspicion of a fistula communicating with the homolateral piriform sinus, was observed. This aspect was in favor of a cystic lymphangioma or a fourth branchial cleft cyst.

**Fig. 3:**
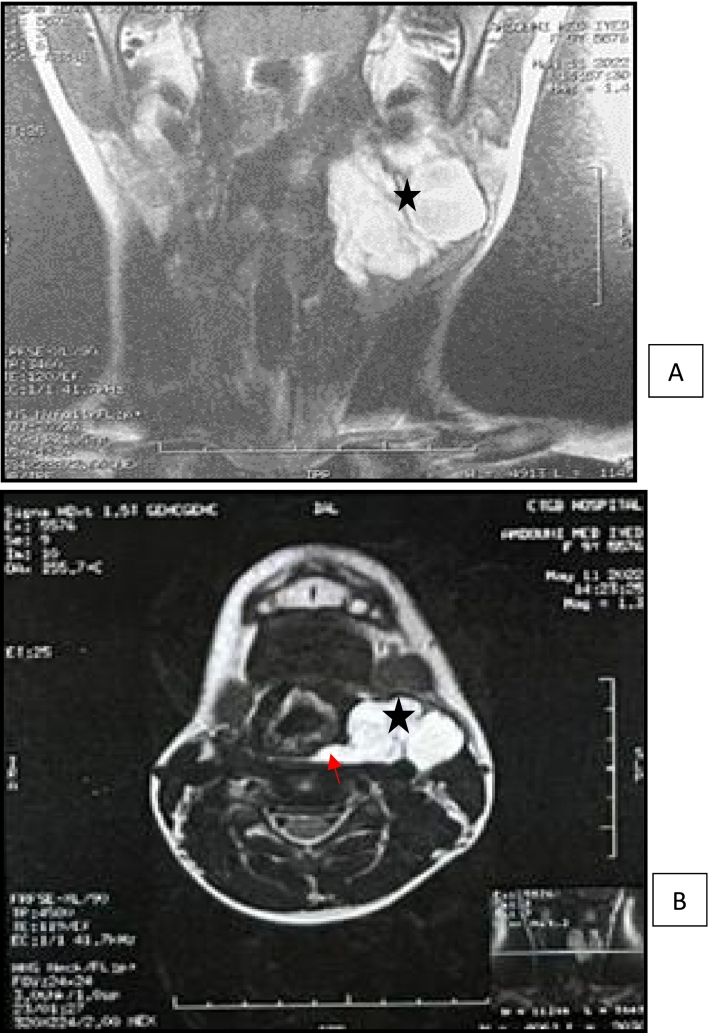
Cervical MRI showing a multiloculated left latero-cervical cyst (black star) on hypersignal on coronal T2 images (A) and axial T2-FATSAT images (B), presenting a retro-pharyngeal extension (red arrow) with no fistula. (For interpretation of the references to colour in this figure legend, the reader is referred to the web version of this article.)

Cervical MRI (Fig. 3) was performed showing the same aspect, confirming retropharyngeal extension and pharyngolaryngeal mass effect, and overruling the presence of an associated fistula, which favors a cystic lymphangioma.

**Fig. 4:**
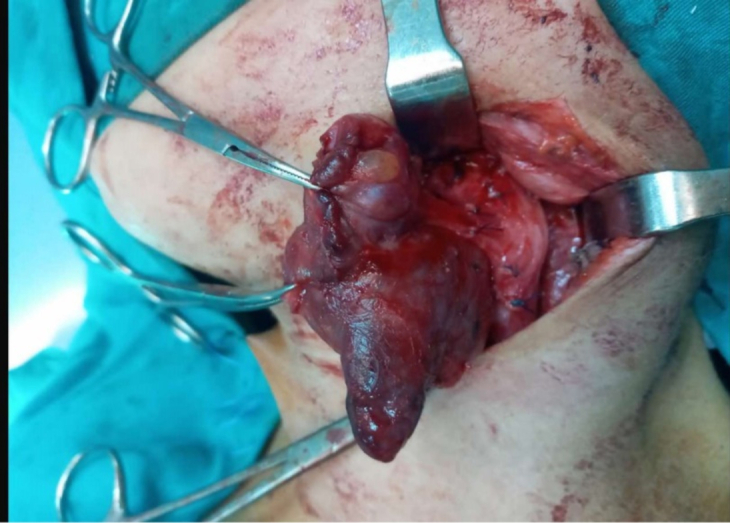
Intraoperative picture showing the lesion (red arrow) underneath the sternocleidomastoid muscle (black arrow). (For interpretation of the references to colour in this figure legend, the reader is referred to the web version of this article.)

The peroperative findings were a multilocular encapsulated cystic mass that extended between the inferior edge of the horizontal branch of the mandible superiorly and the lower part of the thyroid with development towards the back of the pharynx (Fig. 4). The cyst was adherent to the carotid sheath and the internal jugular vein. The lower part of the cyst was located 2 cm above the clavicle. Complete surgical removal of the cyst was performed after separation of the cyst from the neck vessels. Extemporaneous examination was in favor of a cystic lymphangioma. The post-operative course was uneventful, apart from some post-operative dysphagia, which rapidly subsided. The patient was discharged on day three postoperatively. The final anatomopathological study revealed a thymic cyst. No recurrences were observed after a 2-year follow-up.

## 3 Discussion

The thymus derives mainly, from the third pharyngeal pouch [6] or, in some cases, from the fourth [7]. Thymic cysts are benign, rare lesions, with only about 100 cases reported in the literature [3]. They originate from the remnants of the thymo-pharyngeal duct.

To explain the pathogenesis of cervical thymic cysts two theories were developed: first, the persistence of the thymo-pharyngeal duct and second, the degeneration of the Hassal corpuscles [9]. Unilocular cervical thymic cysts are the most common [10] and mediastinal extension is reported in 50 % of cases [11]. A male preponderance is reported [12] with a mean age of onset of 7 years [3].

Thymic cysts are usually asymptomatic [6]. The most common revealing symptom is painless cervical mass. Compressive signs such as dyspnea, dysphagia and dysphonia may be a rarer mode of revelation [7]. Those cysts are often transilluminated and expand with a Valsalva maneuver. More found on the left side of the neck, those cyts are usually located laterally to the thyroid gland, medially medial to the sternocleidomastoid muscle and anteriorly to the carotid sheath [7].

The differential diagnosis includes among other diagnosis: the lymphoma,the thyroglossal duct, the branchial cleft cyst, the cystic hygroma, the teratoma, the cystic thymoma and cystic metastases cysts [8]. Branchial cleft cysts occur more oftenly in the upper half of the neck between the carotid arteries [9].

No preoperative diagnostic method is able to certify the thymic origin of a neck mass, but radiological investigations are helpful. Combining CT and magnetic resonance imaging is essential for diagnosis and management. In the pediatric population, magnetic resonance imaging is preferred to reduce radiation exposure. Both imaging modalities are able to distinguish between cervical thymic cysts, branchial cleft cysts and lymphangiomas, asses mediastinal extension of the lesion and its relation to cervical vessels [6]. However, the radiological differential diagnosis of thymic lesions can be difficult. In fact, infection and bleeding can lead to confusion with solid masses due to the increased density in the CT-scan. On magnetic resonance imaging, thymic cysts are homogeneous with a low or intermediate signal in T1-weighted-images and a high signal in the T2-weighted images [11]. Magnetic resonance imaging shows greater soft tissue detail [15] and can determine the association of putative lesions with the thymus [10].

Fine needle aspiration is not helpful for diagnosis [13]. The definitive diagnosis depends on the histopathological examination [3].

The treatment for thymic cysts consists of complete surgical removal of the intact cyst. In children, it is necessary to ensure the presence of normal thymic tissue in the mediastinum. In fact, this will not cause any problem in the adult, but in children, the thymus plays an important immunological function [13]. Thymectomy during childhood can affect.

A transverse incision over the mass, is done at least 2 fingers below the angle of the mandible to prevent an injury of the marginal mandibular nerve [13]. The incision can be vertical, anterior to the medial border of the sterno-cleido-mastoid muscle. Most cysts are located deeper in the SCM and attached to the carotid sheath [16].

Surgical excision can be difficult because the cyst may be adherent to adjacent structures: the internal jugular vein, the carotid, the vagus, the phrenic, hypoglossal, and laryngeal nerves [2]. The capsule must remain intact [16].

Other therapeutic options include with needle drainage to empty the cyst from its content. However, aspiration of all fluid may be difficult, as some thymic cysts are multiloculated [16]. Temporary observation can be an option, especially in case of infected cysts. But long-term observation is not recommended for persistent or recurrent neck masses without a definitive diagnosis in view of the possibility of infection, the potential for growth, and the possibility of malignancy [16].

Recurrences were not reported after complete excision in the pediatric population [10].

## 4 Conclusions

Although cervical thymic cysts are rare, this diagnosis should be considered in front of any indolent lateral cervical mass. Imaging can be helpful, but the diagnosis is usually made postoperatively. Treatment is complete consists in surgical excision. The prognosis is excellent, and no cases of recurrence have been reported.

## Author contribution

Nadia Romdhane: writing - supervision

Dorra Chiboub: supervision

Selima Jouini; writing - data collection

Ons Kharrat: writing - data collection

Raja Jouini: supervision - data collection

Chiraz Mbarek: supervision

## Informed consent

Written informed consent was obtained from the patient's parents/legal guardian for publication and any accompanying images. A copy of the written consent is available for review by the Editor-in-Chief of this journal on request.

## Ethical approval

This study is exempt from ethical approval at our institution (Habib Thameur Hospital).

## Guarantor

Selima Jouini.

## Funding

The author(s) received no financial support for the research, authorship, or publication of this article.

## Conflict of interest statement

The author(s) declare no potential conflicts of interest concerning the research, authorship, or publication of this article.

## Data Availability

Data is openly available.
